# Socioeconomic inequality in modern contraceptive utilization among reproductive-age women in sub-Saharan African countries: a decomposition analysis

**DOI:** 10.1186/s12913-023-09172-6

**Published:** 2023-02-22

**Authors:** Elsa Awoke Fentie, Desale Bihonegn Asmamaw, Ever Siyoum Shewarega, Wubshet Debebe Negash, Rediet Eristu Teklu, Tewodros Getaneh Alemu, Habitu Birhan Eshetu, Daniel Gashaneh Belay, Fantu Mamo Aragaw, Samrawit Mihret Fetene

**Affiliations:** 1 Department of Reproductive Health, Institute of Public Health, College of Medicine and Health Sciences, University of Gondar, Gondar, Ethiopia; 2 Department of Reproductive Health, School of Public Health, College of Medicine and Health Sciences, Dilla University, Dilla, Ethiopia; 3 Department of Health Systems and Policy, Institute of Public Health, College of Medicine and Health Sciences, University of Gondar, Gondar, Ethiopia; 4 Department of Epidemiology and Biostatistics, Institute of Public Health, College of Medicine and Health Sciences, University of Gondar, Gondar, Ethiopia; 5 Department of Pediatrics and Child Health Nursing, School of Nursing, College of Medicine and Health Sciences, University of Gondar, Gondar, Ethiopia; 6 Department of Health Promotion and Health Behavior, Institute of Public Health, College of Medicine and Health Sciences, University of Gondar, Gondar, Ethiopia; 7 Department of Human Anatomy, College of Medicine and Health Sciences, University of Gondar, Gondar, Ethiopia

**Keywords:** Socioeconomic-related inequality, DHS, Decomposition analysis, Modern contraceptive utilization, Sub-Saharan Africa

## Abstract

**Introduction:**

Family planning services allow individuals to achieve desired birth spacing, family size, and contribute to improved health outcomes for infants, children, women, and families, and prevent unintended pregnancy. Births resulting from unintended pregnancies can have negative consequences Children from unintended pregnancies are more likely to experience poor mental and physical health during childhood. Even though many international organizations work to ensure universal access to sexual and reproductive health services, reproductive health service utilization is concentrated among individuals with rich socioeconomic status. Therefore, this study aimed to assess the presence of socioeconomic inequality in modern contraceptive utilization and its contributors in sub-Saharan African countries.

**Methods:**

A total of 466,282 weighted reproductive-aged women samples from DHS data SSA countries were included in the study. Erreygers normalized concentration index and its concentration curve were used to assess socioeconomic-related inequality in modern contraceptive utilization. Decomposition analysis was performed to identify factors contributing to socioeconomic-related inequality.

**Results:**

The weighted Erreygers normalized concentration index for modern contraceptive utilization was 0.079 with Standard error = 0.0013 (*P* value< 0.0001); indicating that There is small amount but statistically significant pro rich distribution of wealth related in equalities of modern contraceptive utilization among reproductive age women. The decomposition analysis revealed that mass media exposure, wealth index., place of residency, and distance of health facility were the major contributors to the pro-rich socioeconomic inequalities in modern contraceptive utilization.

**Conclusion and recommendation:**

In this study, there is a small amount but statistically significant pro rich distribution of modern contraceptive utilization. Therefore, give priority to modifiable factors such as promoting the accessibility of health facilities, media exposure of the household, and improving their country’s economy to a higher economic level to improve the wealth status of the population.

## Introduction

Family planning is one of the 10 great public health achievements since the twentieth century [1]. The availability of family planning services allows individuals to achieve desired birth spacing and family size, contributes to improved health outcomes for infants, children, women, and families, and prevents unintended pregnancy [2]. These reductions in unintended pregnancies and maternal and newborn mortalities can lead to the attainment of SDG 3 [3].

Moreover, contraceptive use has a number of potential non-health benefits, including increased educational opportunities for women, facilitation of gender equality, social and economic empowerment for reproductive-aged women, sustainable population growth and economic development for countries [4]. However, socioeconomic inequalities in health and health-related services are particularly common in low- and middle-income countries like Sub-Saharan Africa, where the poor are disproportionately affected [5].

Among the 1.9 billion women of reproductive age group (15–49 years) worldwide in 2019, 1.1 billion need family planning; of these, 842 million are using contraceptive methods, and 270 million have an unmet need for contraception, and Among contraceptive users, the vast majority (45%) use modern methods [6, 7]. The use of contraception among women of reproductive age in sub-Saharan Africa increased from 13% in 1990 to 29% in 2019 [8]. Previous studies have documented that modern contraceptive utilization is significantly associated with economic status, age, educational status of the woman and husband, marital status, residency, access to the health facility, media exposure, knowledge of modern contraceptive utilization, spousal decision-making, couple discussion about family planning, contraceptive misconception, and parity [9–15].

Not using contraceptive methods result in unintended pregnancy and births resulting from unintended pregnancies can have negative consequences including birth defects, andlow birth weight [16]. Furthermore, each unintended pregnancy may put women at risk for significant morbidity and mortality due to unsafe abortion, poverty, malnutrition, and lack of health care [17]. the consequences associated with unintended pregnancies are greater for teen parents and their children [18].

Although different governmental and non-governmental organizations work to reduce the global maternal mortality ratio to less than 70 per 100,000 live births and end all preventable deaths under 5 years of age by 2030, respectively, by ensuring sexual and reproductive health services for all, the progress is not adequate, especially in sub-Sharan African countries.

There is also limited information about socioeconomic-related inequality in modern contraceptive utilization in sub-Saharan Africa. Therefore, this study aimed to assess the presence of socioeconomic inequality in modern contraceptive utilization and its contributors in sub-Saharan African countries using recent demographic and health surveys using decomposition Analysis. This will help countries to ensure their disadvantaged populations are not left behind and help policymakers to narrow the disparity of modern contraceptive utilization by wealth status.

## Methods

### Study design, setting, and period

The data source for this study was the recent standard Demographic health survey data of Sub-Saharan African countries conducted within 10 years (2010–2020), which was a crossectional study conducted every five-year interval (Table 1). The DHS is a national survey that collects information on basic health indicators such as mortality, morbidity, family planning service use, fertility, and mother and child health. The sub-Saharan is the area in the continent of Africa that lies south of the Sahara and consists of four geographically distinct regions namely Eastern Africa, Central Africa, Western Africa, and Southern Africa.

**Table 1:** Sample size determination of modern contraceptive utilization and factor associated with it among reproductive age women in each sub-Saharan Africa: based on 2010–2020 DHS

Region	Country	DHS year	Weighted sample
East Africa	Burundi	2016/17	17,269
	Comoros	2012	5329
	Ethiopia	2016	15,683
	Kenya	2014	31,079
	Malawi	2015/16	24,562
	Mozambique	2011	13,745
	Rwanda	2014/15	14,634
	Tanzania	2015/16	13,266
	Uganda	2016	18,506
	Zambia	2018	13,683
	Zimbabwe	2015	9955
Central Africa	Angola	2015/16	14,379
	Cameroon	2018	13,616
	Chad	2014/15	17,719
	DR Congo	2013/14	18,827
	Congo	2011/12	10,819
	Gabon	2012	8422
Western Africa	Benin	2017/18	15,928
	Burkina Faso	2010	17,087
	Ivory Coast	2011/12	10,060
	Gambia	2013	11,865
	Ghana	2014	9396
	Guinea	2018	10,874
	Liberia	2019/20	8065
	Mali	2018	10,519
	Niger	2012	11,160
	Nigeria	2018	41,821
	Senegal	2019	8649
	Sierra Leone	2019	15,574
	Togo	2013/14	9480
South Africa	Lesotho	2014	6621
	Namibia	2013	9176
	South Africa	2016	8514

### Population

The source population was all reproductive-age women across 33 Sub-Saharan African countries. Whereas the study population was reproductive-age women in the selected Enumeration Areas (EAs) and the mother was interviewed for the survey in each country.

### Inclusion criteria

All reproductive-age women in the selected EAs in each SSA country were included in this study.

### Exclusion criteria

Five countries that did not have a survey report after the 2010/2011 survey year were excluded due to the recent updates: Central Africa Republic, Eswatini, Sao Tome Principe, Madagascar, and Sudan. As well as three Sub-Saharan Countries (Botswana, Mauritania, and Eritrea) were excluded due to the dataset not being publicly available.

### Sampling procedures and sample size

A total of 47 countries are located in sub-Saharan Africa. Of these countries, only 33 countries had Demographic and Health Survey Report after 2010. A two-stage stratified cluster sampling technique was employed in DHS data. First, clusters/enumeration areas (EAs) were randomly selected from the sampling frame (i.e. are usually developed from the available latest national census). Second, systematic random sampling was conducted on households listed in each cluster or EA. Finally, interviews were conducted in selected households with target populations (women aged 15–49 and men aged 15–64) [19]. Weighted values were used to restore the representativeness of the sample data and were calculated from Individual Record (IR) DHS datasets. Finally, a total weighted sample of 466,282 reproductive-aged women was included from all 33 countries in sub-Saharan African countries (Table 1).

### Study variables

#### Dependent variables

Socioeconomic-related inequality in current modern contraceptive use was the outcome variable in this study. Current modern contraceptive utilization was a composite variable. If women reported the use of one of the following methods: female sterilization, male sterilization, the contraceptive pill, intrauterine contraceptive device (IUD), injectables (Depo Provera), implants, female condom, male condom, diaphragm, contraceptive foam and contraceptive jelly, lactational amenorrhea method (LAM), standard days method (SDM), country-specific modern methods and respondent-mentioned other modern contraceptive methods (including cervical cap, contraceptive sponge, and others were considered as currently using modern contraceptive while if a woman didn’t use none of the above modern contraceptive methods were considered as not using modern contraceptive currently [19]. The socioeconomic-related inequality of current modern contraceptive utilization can be expressed as the covariance between current modern contraceptive use and the measurement for living standards distribution (wealth index). Then, it was classified into either pro-poor, pro-rich, or no inequality. When the curve lies above the line of equality (when the ECI takes a negative value) the health variable in this case modern contraceptive use is concentrated among the poor (pro-poor). However, the ECI value can be positive, the curve will be below the line of equality indicating the health variable is concentrated among the rich (pro-rich). The ECI will be zero in the case when there is no socioeconomic-related inequality, the concentration curve lies at a 45-degree line (the line of perfect equality).

#### Independent variables

Women’s age, educational level, wealth index, sex of household head, mass media exposure, place of residence, husbands’ educational level, current working status, parity, modern contraceptive knowledge, women’s involvement on decision-making of maternal health, −sub-regions in SSA and distance of health facility were incorporated as explanatory variables. The socioeconomic status was measured using the wealth index from DHS data sets. In the DHS data, the wealth index was constructed using principal component analysis and then categorized as poorest (quintile 1), poorer (quintile 2), middle (quintile 3), richer (quintile 4), richest (quintile 5) [20]. media exposure (media exposure was created from the three variables: watching television, listening radio, and reading a newspaper, and labeled as yes if a woman has exposure to either of the three media sources or no if a woman has exposure to none of them [21].

### Data management and statistical analysis

This study was performed based on the DHS data obtained from the official DHS measure website. DHS data in STATA format then cleaned, transformed, and append to produce favorable variables for the analysis. STATA 16 software was used to generate both descriptive and analytic statistics of the appended 33 countries’ data. Sampling weight was used throughout the analyses to adjust for the unequal probability of selection of the sample and the possible differences in response rates. The frequency with percent was used to indicate the distribution of respondents’ background characteristics and *p*-values were computed using Pearson’s chi-squared test.

The study used a concentration curve to identify whether socioeconomic inequality in some health variables exists and to examine whether it is more pronounced at one point than another. Besides, the study also used a concentration index [22] to quantify and compare the degree of socio-economic-related inequality in a health variable [23, 24]. The concentration index is twice the area between the concentration curve and the line of equity with the range of − 1 to + 1 and the sign indicates the direction of the relationship between current modern contraceptive utilization and the distribution of living standards (wealth status) (Accordingly, CI = 0 indicated the distribution was proportionate, CI = 1 displayed that the richest person had all of the health variables, whereas CI = − 1 indicated that the poorest person had all of the health variables) [25, 26] But the outcome variable in the present study is binary (use/not use modern contraceptive), the bounds of C depend on the mean (μ) of the outcome variable and do not vary between 1 and-1. Thus, the bounds of C vary between μ–1 (lower bound) and 1–μ (upper bound) so the present study used Erreygers normalized concentration index (ECI) which is a modified version of the concentration index [27].

Mathematically, ECI can be defined as:

$$\textrm{ECI}={4}^{\ast }{\mu}^{\ast}\textrm{CI}\left(\textrm{y}\right).$$

Where ECI is Erreygers concentration index, CI(y) is the generalized concentration index and μ is the mean of the health variable, current modern contraceptive utilization. Then, the ECI with the standard error (SE) was reported in this study.

To graphically show the socioeconomic-related inequality in current modern contraceptive utilization, Concentration curves show the cumulative percentage of the current modern contraceptive use (y-axis) against the cumulative share of the population ranked by living standards beginning with the poorest and ending with the richest (x-axis) [26]. The ECI would be a 45^0^-line running from the bottom left-hand corner to the top right-hand corner indicating the absence of Inequality (ECI = 0). Furthermore, the concentration curve lying above and below the equality line (45^0^) indicated that the health variable is disproportionately concentrated between poor(pro-poor or ECI < 0) and rich(pro-rich or ECI > 0), respectively [26, 28]. Visual inspection of a concentration curve can give information regarding whether the concentration curve lies above or below the line of equality. To assess the statistical significance of the difference between the concentration curve and the line of perfect equality (45-degree or diagonal line), the ECI with its *p*-value was calculated.

To identify the relative contribution of various factors to socioeconomic-related inequality in current modern contraceptive utilization, a decomposition of the ECI was performed [26, 28, 29]. For any linear additive regression model of health outcome (y) [26],

$$y=\mu +{\Sigma}_k{\beta}_k{X}_k+\in$$

The concentration index for y, CI, is given as:

$$y={\sum}_k\left(\frac{\beta_k{\overline{X}}_k}{\mu}\right){C}_k+\frac{gc_{\in }}{\mu }$$

Where “y” is the health outcome variable (in this case socioeconomic related inequality of modern contraceptive utilization), *X*_*k*_ is a set of the socioeconomic determinants of the health outcome, α is the intercept, *β*_*k*_ is the coefficient of *X*_*k*_, μ is the mean of y, ${\overline{X}}_k$ is the mean of *X*_*k*_, *C*_*k*_ is the CI for *X*_*k*_, *gc*_∈_ is the generalized CI for the error term (∈), $\frac{\beta_k{\overline{X}}_k}{\mu }$ is the elasticity of y with respect to ${\overline{X}}_k$ [29, 30].

## Result

### Socio-demographic characteristics of study participants

A total weighted 466,282 reproductive-aged women were included in this study. 21.11% of women were in the age group of 15–19 years, with a median age of 27 (IQR: 19) years. More than three-fifths of women (69.98%) had formal education and 61.14% of the women were not working. Near to three-fifths (59.53%) of the respondents were rural inhabitants. Moreover, 66.79% of husbands decided about maternal health alone (Table 2).

**Table 2:** Socio-demographic characteristics of the reproductive age women in a study of socio-economic inequality of modern contraceptive utilization in Sub-Saharan Africa: based on 2010–2020 DHS

Variable	Category	Weighted frequency	Weighted %
Regions of SSA	East	110,437	23.68
	Central	68,644	14.72
	South	105,964	22.73
	West	181,237	38.87
Age	15–19	98,439	21.11
	20–24	85,825	18.41
	25–29	81,861	17.56
	30–34	66,999	14.37
	35–39	57,064	12.24
	40–44	41,868	8.98
	45–49	34,226	7.34
Residence	Urban	188,695	40.47
	Rural	277,587	59.53
Educational level	No education	139,997	30.02
	Primary	146,903	31.51
	Secondary	153,774	32.98
	Higher	25,608	5.49
Occupation	Not working	179,840	38.57
	Working	286,442	61.43
Husband educational level	no education	275,737	59.14
	Primary	79,979	17.15
	Secondary	86,100	18.47
	Higher	24,466	5.25
Sex of household head	Male	336,879	72.25
	Female	129,403	27.75
Mass media exposure	No	139,512	29.92
	Yes	326,770	70.08
No of living children	No children	130,961	28.09
	1–2	140,071	30.04
	3–4	104,694	22.45
	≥5	90,556	19.42
**Distance of health facility**	Not big problem	272,424	62.79
	Big problem	161,337	37.18
**Decision on maternal health**	Respondent	48,513	10.4
	Both	106,329	22.8
	Husband alone	311,440	66.79
**Wealth index**	Poorest	81,230	17.42
	Poorer	86,095	18.46
	Middle	89,656	19.23
	Richer	98,019	21.02
	Richest	111,281	23.87

### The pooled magnitude of modern contraceptive use among reproductive-age women

**Fig. 1:**
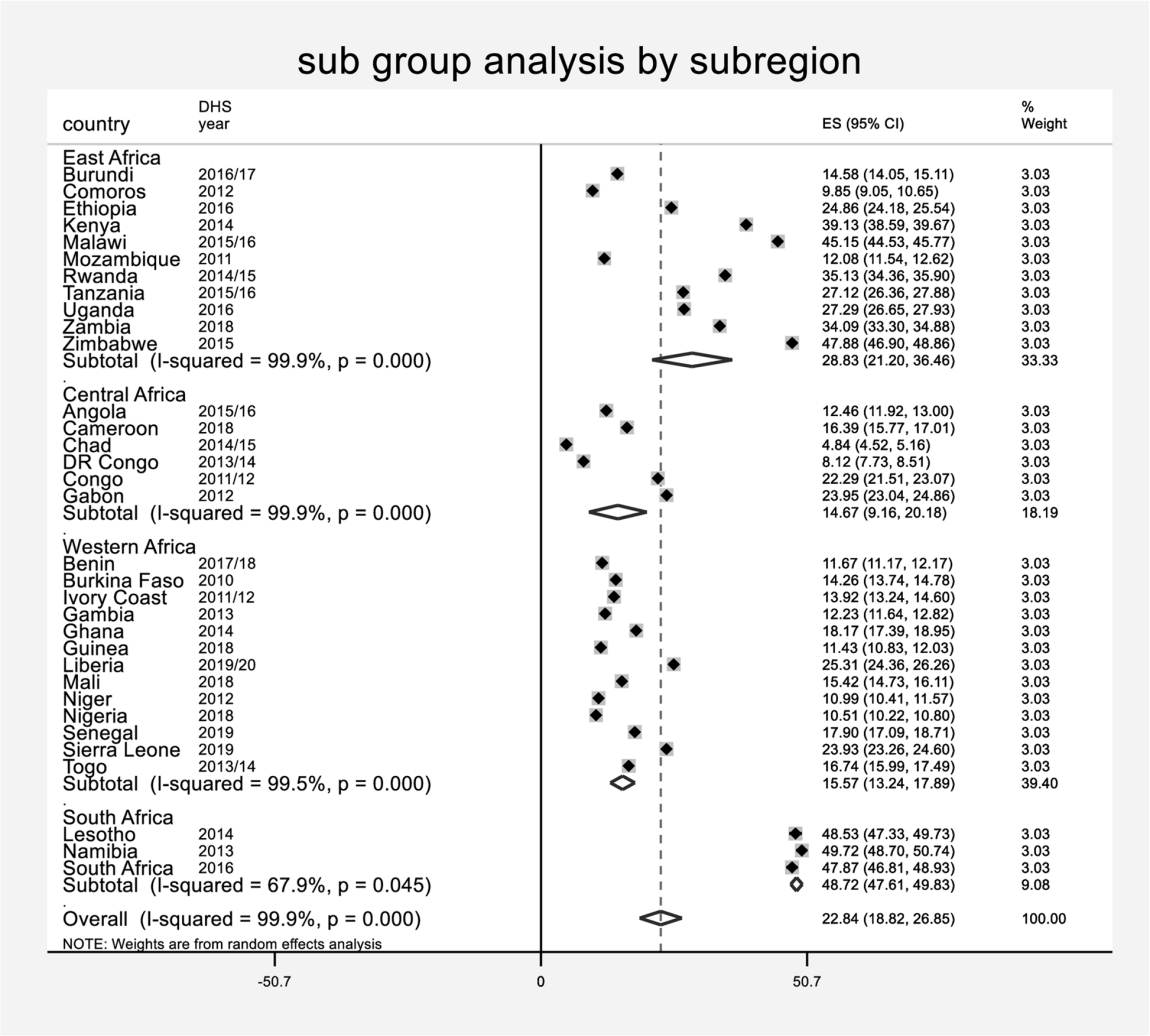
The Forest plot showed that pooled magnitude modern contraceptive use among reproductive age women in SSA based on Sub region

The overall pooled estimate of modern contraceptive use among reproductive-age women in Sub-Saharan African countries was 22.84 (95%CI: 18.82, 26.85%), with I2 = 99.9% and ranging from 4.84% in Chad to 49.72% in Nambia. Moreover, the pooled magnitude of modern contraceptive use across the sub-region was determined. The pooled estimate of modern contraceptive use in East African countries was 28.83% (95%CI: 21.20, 36.46%), Central African countries 14.67% (95%CI: 9.16 20.18%), Western African countries (95%CI: 13.24,17.89%), and 48.72% across South African countries (95%CI: 47.61, 49.83%) (Fig. 1).

### Wealth-related inequality in modern contraceptive utilization

**Fig. 2:**
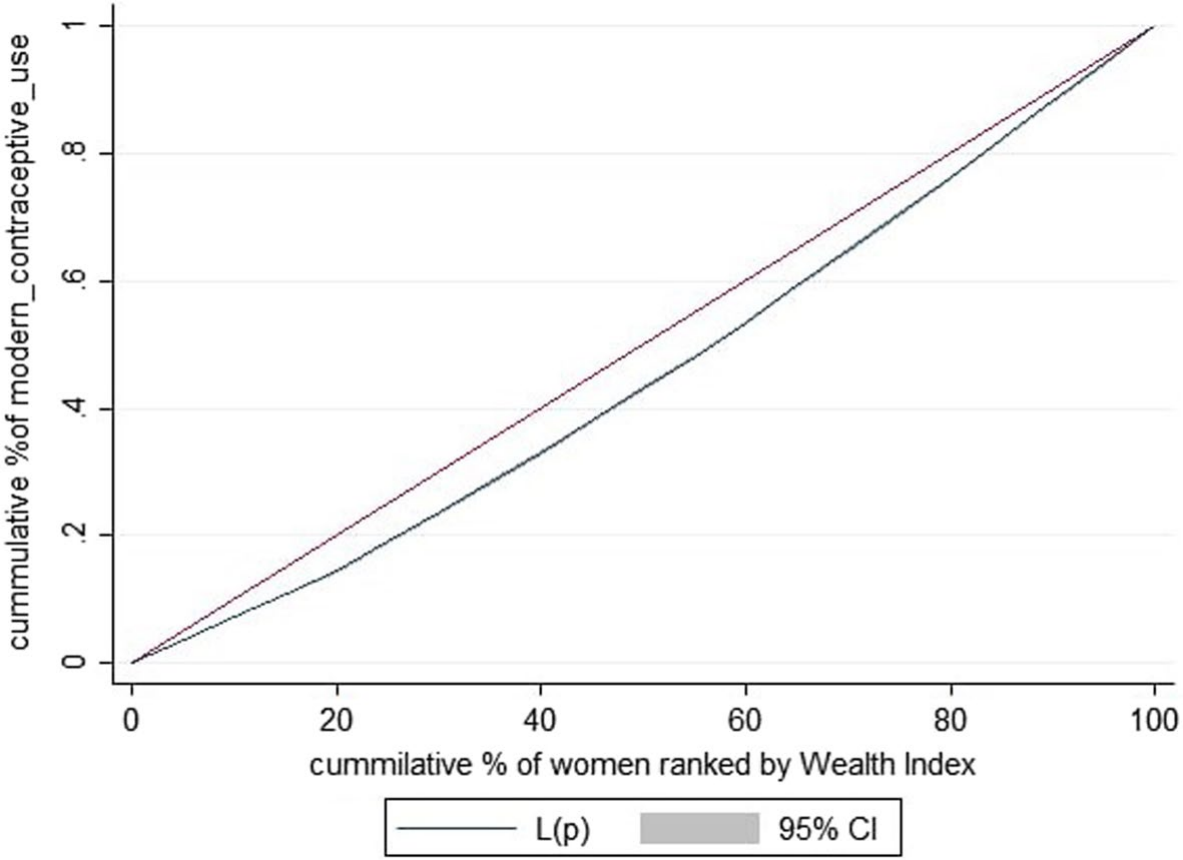
Concentration curve for modern contraceptive utilization in Sub-Saharan Africa

The weighted Erreygers normalized concentration index (ECI) for modern contraceptive utilization was 0.079 with Standard error = 0.0013 (*P* value< 0.0001) (Fig. 1). This revealed that There is small amount but statistically significant pro rich distribution of wealth related in equalities of modern contraceptive utilization reproductive age women. The concentration index is twice the area between the concentration curve and the diagonal line (Fig. 2). Then when multiplying the C by 75 [31] (0.079*75) =5.9, which showed that 6% of the modern contraceptive utilization would need to be redistributed from the richer half to the poorer half of the population to arrive at a distribution with an index value of zero (perfect equality).

Similarly, the concentration curve showed that the concentration graph of modern contraceptive utilization was below the line of equality which indicated that the distribution of modern contraceptive use was concentrated in rich households (pro-rich distribution) (Fig. 2).

### Decomposing the socioeconomic-related inequality in modern contraceptive utilization

After the concentration index and curve were assessed and showed income-related inequality to modern contraceptive utilization. A decomposition analysis was conducted based on Erreygers normalized concentration index to verify how much of the measured socioeconomic inequality in modern contraceptives was due to wealth quintiles and other variables. The analysis shows the contributions of individual variables to the overall socioeconomic inequality of modern contraceptive use. To understand the factors that contribute to socio-economic inequality, coefficient and its significant level, elasticity, concentration index, and percent contribution were calculated.

Nearly one-fourth (24.17%) of the pro-rich inequalities in modern contraceptive utilization among reproductive-age women is explained by the residents. Having media exposure also explained 23.93% of the pro-rich wealth-related inequality for modern contraceptive utilization among reproductive-age women. The other 13.92% of the estimated pro-rich inequalities in modern contraceptive utilization are explained by the distance of health facility (Table 3).

**Table 3:** Contributing factors of socio-economic inequality in modern contraceptive utilization in Sub-Sharan Africa

Variables	Category	Coefficient	Elasticity	Concentration index	Absolute contribution	% Contribution
Regions of SSA	East					
	Central*	−0.1355	−0.0808	−0.0094	0.0008	1.01
	south *	0.0425	0.0385	−0.0024	−0.0001	−0.13
	West *	−0.0980	− 0.1521	− 0.0042	0.0006	0.76
Subtotal					0.0013	**1.64**
Age	15–19					
	20–24*	0.1445	0.1061	0.0270	0.0029	3.67
	25–29*	0.1714	0.1201	0.0208	0.0025	3.16
	30–34*	0.1794	0.1029	0.0043	0.0004	0.50
	35–39*	0.1747	0.0853	−0.0185	−0.0016	−2.03
	40–44*	0.1432	0.0513	−0.0409	− 0.0021	−2.66
	45–49*	0.0641	0.0188	−0.0647	−0.0012	−1.52
Subtotal					0.0009	**1.12**
Residence	Urban					
	Rural *	−0.0113	−0.0269	−0.7101	0.0191	24.17
Currently working	No					
	Yes *	0.0195	0.1257	−0.0514	−0.0065	−8.22
Marital status	Single					
	Married*	0.0310	0.0784	−0.1512	−0.0119	−15.06
	Divorced/widowed*	0.0232	0.0081	−0.0443	−0.0004	− 0.51
Sub total					−0.0123	**−15.57**
Educational level	no education					
	Primary *	0.0488	0.0335	−0.1822	−0.0061	−7.72
	Secondary*	0.0257	0.0189	0.1517	0.0029	3.67
	Higher *	0.0220	0.0046	0.5228	0.0024	3.04
Sub total					−0.0008	**−1.01**
Sex of household head	Male					
	Female*	−0.0052	−0.0057	0.0239	−0.0001	− 0.13
Mass media exposure	No					
	Yes *	0.0322	0.0903	0.4677	0.0422	53.45
Distance of health facility	Not big problem					
	Big problem*	−0.0177	−0.0266	−0.3954	0.011	13.92
Decision on maternal health	Respondent					
	Both *	−0.0117	−0.0107	−0.0070	0.0001	0.13
	Husband alone*	−0.0521	−0.1392	0.0058	−0.0008	−1.01
Sub total					−0.0007	**−0.88**
Wealth index	Poorest					
	Poorer *	0.0203	0.0150	−0.5725	−0.0086	−10.89
	Middle *	0.0295	0.0227	−0.1110	−0.0025	−3.16
	Richer *	0.0370	0.0311	0.3962	0.0123	15.57
	Richest *	0.0248	0.0237	0.824	0.0195	24.72
Sub total					0.0249	**26.24**

* = *p* value< 0.05

## Discussion

This study aimed to assess the socioeconomic inequality in modern contraceptive use and its contributors among reproductive-aged women in sub-Saharan Africa. According to this study, modern contraceptive use in SSA was disproportionately concentrated among rich households. Evidence has also supported that the uptake of maternal health services is inequitable to the disadvantage of the poor and higher utilization of maternal health services by richer women [30–33]. This implied that economically disadvantaged women had limited utilization of modern contraceptives, which had a great impact on women’s ability to enjoy universal access to reproductive health services. Therefore, strengthening inter-sectoral collaboration among development sectors is crucial to reduce poverty in order to improve maternal health and promote equity.

In decomposition analysis, several factors were contributing to the pro-rich socioeconomic inequalities in modern contraceptive utilization where the distance of health facility, wealth index, residency, and mass media exposure were the major contributors to this inequality.

It was found that media exposure was the major and important contributor to the overall socioeconomic inequality in modern contraceptive utilization (53.42%). This finding is in line with studies done in-sub-Saharan African countries [33], Ethiopia [30], and Afghanistan [34]. This might be due to mass media can expose people to information concerning health and this may improve the knowledge and attitude of women towards health service utilization [35].

Following mass media exposure, the wealth index was also a significant contributor to the overall socioeconomic inequality in modern contraceptive utilization (26.24%). Previous studies had also revealed that wealth is the main determinant factor for maternal health service utilization [30, 34, 36]. The possible reasons might be that women who had a better wealth index may help access health care or a better wealth index may reduce the difficulties of obtaining money to access health care [37].

This study also revealed that residency was another contributor to the socio-economic inequality in modern contraceptive utilization (24.17%). Previous studies also highlighted that residency had strong positive relationships with health service access and maternal health service utilization [33, 38, 39]. The possible reason for this finding could be due to women in rural areas had relatively poor healthcare-seeking behavior and low access to health information [40]. Moreover, rural women had poor service accessibility and there are also sociocultural issues related to lower male involvement and support for women’s healthcare access [41].

Regarding with distance of health facilities. it had significantly contributed to socioeconomic inequality for modern contraceptive utilization. This might be due to the distance of the health facility imposing an extra cost for transportation to reach to a health facility as well as the lack of availability of transportation making women fail to go to the health facility to utilize health services [42].

The main strength of this study was the use of the weighted nationally representative data of each Sub-Saharan African country with a large sample which makes it representative at Sub-Saharan and regional levels. Moreover, the ECI and curve and wag staff decomposition analysis are appropriate statistical models to show the direction and degree of socioeconomic inequality of modern contraceptive use between the poorest to the richest household. First, due to the cross-section nature of the data, the findings cannot provide information on temporal relationships among the variables as a result casual inference couldn’t be drawn. Since the data were collected cross-sectionally at different points in time self-reported interviews would be prone to social desirability bias.

## Conclusion and recommendation

The proportion of modern contraceptive utilization among reproductive-age women in sub-Saharan Africa was relatively low. There is small amount but statistically significant pro rich distribution of wealth related in equalities of modern contraceptive utilization among reproductive age women. Wealth index, place of residency, the distance of health facility, and mass media exposure were the major contributors to pro-rich socioeconomic inequalities of modern contraceptive utilization. Therefore, targeting disadvantaged women and contributors will help to alleviate these inequalities and enhance universal health coverage.

To increase modern contraceptive use among reproductive-age women in sub-Saharan Africa, policymakers and other stakeholders should work together with other sectors, and give priority to modifiable factors such as promoting the accessibility of health facilities, and media exposure of the household. For those SSA countries with lower income status needed long-term plans to improve their country’s economy to a higher economic level and to improve the wealth index of individual households. Interventions to improve modern contraceptive use also need balance by supporting marginalized groups such as rural residents.

## Data Availability

The datasets used and/or analyzed for this study are available from the Demographic and Health Surveys (DHS) Program (https://dhsprogram.com/Data/).

## Abbreviations

CI: Concentration Index
DHS: Demographic and Health Survey
ECI: Erreygers Concentration Index
DHS: Demographic and Health Survey
SSA: Sub-Sharan Africa
SDG: Sustainable Development Goal
WHO: World Health Organization
